# SDF vs resin sealants in preventing caries among high-risk children: a 2-year randomized trial

**DOI:** 10.1590/0103-644020266778

**Published:** 2026-05-01

**Authors:** Gabrielly Fernandes Machado, Daniele Morais Dias, Izabella Barbosa Fernandes, Kaio H. Soares, Dhelfeson Willya Douglas de Oliveira, Patricia Furtado Gonçalves, Rodrigo Galo

**Affiliations:** 1 Department of Dentistry, Faculty of Biological and Health Sciences, Federal University of the Jequitinhonha and Mucuri Valleys, Diamantina, Brazil; 2 Department of Dentistry, Ribeirão Preto School of Dentistry, University of São Paulo, São Paulo, Brazil; 3 Department of Pediatric and Adolescent Oral Health, School of Dentistry, Federal University of Minas Gerais, Belo Horizonte, Brazil

**Keywords:** Child, Dental Caries, Prevention, Resin Sealant, Silver Diamine Fluoride

## Abstract

This study was a parallel, double-blind, randomized clinical trial that aimed to evaluate the effect of silver diamine fluoride (SDF) on the prevention of dental caries in the first permanent molars of children. A total of 68 students aged 6 to 9 years from a public school in Diamantina, Brazil, participated. All children were classified as high caries risk and had at least one fully erupted molar without restorations or sealants. The participants were randomly divided in a 1:1 ratio to receive either SDF or resin sealant. The main outcome was the incidence of new carious lesions after 24 months. Secondary outcomes included plaque accumulation, gingival bleeding, oral health-related quality of life (OHRQoL, CPQ-8-10), caregiver satisfaction, and discomfort reported by the children using the Wong-Baker scale. Sociodemographic and health information were collected using questionnaires, dietary diaries, and clinical examinations in accordance with ICDAS-II criteria. Statistical analysis was performed using SPSS 22.0, employing Chi-square, Mann-Whitney, and Wilcoxon tests. Most families had low incomes, and mothers were usually responsible for the children's daily care. After 2 years, the SDF group showed a significant reduction in plaque compared with baseline, whereas the sealant group showed a small, non-significant increase. No significant difference in gingival bleeding was observed between groups. Both interventions were well tolerated, although the dark staining of the SDF-treated teeth was clearly visible. In conclusion, SDF demonstrated good preventive performance and acceptance among children at

## Resumo

Este estudo foi um ensaio clínico randomizado, paralelo e duplo-cego, que teve como objetivo avaliar o efeito do fluoreto diamino de prata (FDP) na prevenção de cárie dentária nos primeiros molares permanentes de crianças. Participaram 68 escolares, com idades entre 6 e 9 anos, de uma escola pública de Diamantina, Minas Gerais, Brasil. Todas as crianças foram classificadas como de alto risco para cárie e possuíam pelo menos um molar totalmente erupcionado, sem restaurações ou selantes. Os participantes foram distribuídos aleatoriamente, na proporção de 1:1, para receber aplicação de FDP ou selante de resina. O desfecho principal foi a ocorrência de novas lesões de cárie após 24 meses. Os desfechos secundários incluíram acúmulo de biofilme, sangramento gengival, qualidade de vida relacionada à saúde bucal (OHRQoL, CPQ-8-10), satisfação dos cuidadores e desconforto relatado pelas crianças, avaliado pela escala de Wong-Baker. As informações sociodemográficas e de saúde foram coletadas por meio de questionários, diários alimentares e exames clínicos realizados conforme os critérios do ICDAS-II. A análise estatística foi conduzida no software SPSS 22.0, utilizando os testes do Qui-quadrado, do Mann-Whitney e do Wilcoxon. A maioria das famílias tinha baixa renda, e as mães eram, na maior parte dos casos, as principais responsáveis pelos cuidados diários das crianças. Após dois anos, observou-se uma redução significativa do biofilme no grupo tratado com FDP em comparação ao valor inicial, enquanto o grupo do selante apresentou um pequeno aumento, sem significância estatística. Não houve diferença significativa no sangramento gengival entre os grupos. Ambas as intervenções foram bem toleradas, embora o escurecimento dos dentes tratados com FDP tenha sido claramente perceptível. Em conclusão, o FDP apresentou bom desempenho preventivo e aceitação entre crianças com alto risco de cárie, sendo especialmente útil em contextos onde o acesso aos cuidados odontológicos ainda é limitado.

**Figure:**
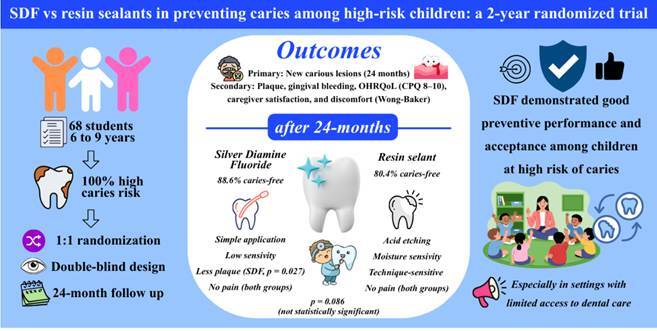

## Introduction

Dental caries continues to be a persistent public health problem that affects not only adults but also many children worldwide ^1^ ^,^ ^2^. Even with continuous preventive efforts and better awareness, the overall decline in caries prevalence has been modest in recent years ^3^ ^,^ ^4^. The condition results from a complex interaction of biological, environmental, and behavioral factors, and dental care has gradually moved toward more conservative and preventive approaches ^5^ ^,^ ^6^ ^,^ ^7^ ^).^ The current focus is not only on treating lesions but also on preserving the tooth structure and preventing new ones.

Among preventive strategies, pit-and-fissure sealants are widely used to protect the occlusal surfaces of permanent molars. They act as a physical barrier, helping prevent bacterial accumulation and food retention in pits and fissures ^8^ ^,^ ^9^. However, the long-term success of sealants depends heavily on their retention and integrity, which may vary depending on the operator's clinical skills, the type of material used, and each child's individual characteristics ^10^ ^,^ ^11^. Additionally, proper isolation and moisture control can be particularly challenging in public health settings or when treating young children ^12^.

Silver diamine fluoride (SDF), especially in its 38% formulation (44,800 ppm fluoride), has recently gained attention as an alternative approach for caries prevention ^13^. It combines the remineralizing action of fluoride with the antibacterial properties of silver ^14^ ^,^ ^15^. When applied to the tooth surface, SDF reacts with hydroxyapatite to form calcium fluoride, silver phosphate, and silver-protein compounds, which help arrest active lesions and increase mineral density ^16^ ^,^ ^17^. Because it is easy to apply, affordable, and requires minimal equipment, SDF has become a practical choice for communities and regions with limited access to dental care ^18^ ^,^ ^19^.

However, SDF is not free from limitations. The dark staining it leaves on treated areas can be a concern for patients and caregivers, especially when visible teeth are affected ^20^. Acceptance varies, and it is essential to consider whether families are willing to make an aesthetic compromise in exchange for improved prevention.

Considering these aspects, comparing SDF with conventional resin sealants is clinically and scientifically relevant. This type of comparison can help determine whether a simple, low-cost material such as SDF can provide similar preventive benefits to those obtained with resin sealants in children at high risk of caries. This rationale guided the design and planning of the present clinical trial.

## Materials and Methods

The present study was approved by the Research Ethics Committee on Human Subjects at the Federal University of Vales do Jequitinhonha and Mucuri (UFVJM), under protocol number 3.359.285. All parents received an introductory letter along with the Free and Informed Consent Form, ensuring their awareness and agreement to participate, as well as the confidentiality and anonymity of their children.

This blinded, randomized clinical trial was conducted in Diamantina, a city in the northeastern region of Minas Gerais state, located in southeastern Brazil. To minimize potential biases and ensure the integrity of the results, double-blinding was rigorously implemented. Specifically, participants (children and their parents/caregivers) were blinded to their assigned intervention, being informed only that they would receive one of two preventive treatments (SDF or resin sealant). Furthermore, all clinical examiners responsible for assessing the primary and secondary outcomes (incidence of new carious lesions, plaque accumulation, gingival bleeding, oral health-related quality of life, and discomfort) were blinded to the treatment group allocation, ensuring they were unaware of which treatment each child had received during evaluations. Finally, the statistician responsible for all data analyses remained blinded to the treatment group allocation (i.e., the identity of the SDF group versus the sealant group) until all statistical analyses were completed.

The study included volunteers who met the following inclusion criteria: children aged 6 to 9 years, enrolled in a school in Diamantina, with at least one fully erupted permanent first molar, no restorations and/or sealants, high risk of developing dental caries, and parental consent for participation. For each eligible child, one first permanent molar was selected and randomly assigned for treatment.

The classification of 'high caries risk' was determined through a multi-factorial assessment that integrated clinical findings from the initial screening with socioeconomic and behavioral indicators. Specifically, participants were considered at high caries risk if they exhibited: ^1^ low socioeconomic status, commonly characterized by family income ranging from 1 to less than 2 minimum wages (as identified from questionnaires and consistent with the study's recruitment from a public school in an underserved region); ^2^ clinical signs of active caries lesions (in primary or permanent dentition, excluding the study molars, assessed using ICDAS-II criteria), presence of restorations, or poor oral hygiene (indicated by visible plaque and gingival inflammation indices) during the initial screening; and/or ^3^ reported infrequent dental visits or high sugar consumption based on preliminary questionnaires and dietary diaries. This comprehensive approach ensured the selection of a cohort genuinely vulnerable to caries progression. Exclusion criteria were children with physical or neurological impairments, respiratory or chronic diseases, allergies to dental materials, or those who declined to participate in the study, uncooperative behavior that prevented the safe and effective application of interventions, current systemic or professional topical fluoride therapy, or the presence of fixed orthodontic appliances.

The sample size was calculated based on the estimation of two proportions, using a success rate of 65% for Silver Diamine Fluoride (SDF) in preventing carious lesions in permanent first molars over three years (DOS SANTOS et al., 2011) and 91.4% for resin-based pit and fissure sealants over three years (HILGERT et al., 2015). Although our trial lasted 2 years, these 3-year success rates were selected as a conservative approach to ensure adequate statistical power, since longer-term data often impose more stringent conditions for detecting treatment differences. The difference to be detected was 26.4%, with a confidence level of 95% (α = 0.05) and a test power of 80% (1 - β). These parameters were used to calculate the sample size in Epi Info (Centers for Disease Control and Prevention, Atlanta, GA, USA), resulting in a minimum of 36 permanent first molars per group in the study. It is crucial to clarify that, for this study, each participating child contributed only one first permanent molar to the primary outcome assessment. Therefore, the '36 permanent first molars per group' directly corresponds to 36 children per group, eliminating any unit-of-analysis error related to multiple teeth from the same subject.

Before data collection, the evaluators underwent theoretical training for caries assessment according to the criteria of the International Caries Detection and Assessment System (ICDAS) (Pitts N., "ICDAS", 2004), as well as training on the criteria for evaluating gingivitis (Mühlemann et al., 1971) and dental plaque (Ainamo et al., 1975). Subsequently, practical training was conducted with 10% of the total number of children.

The examiner performed the clinical calibration examination, and a researcher was used to ensure inter-examiner agreement, as assessed by the Kappa coefficient. Calibration involved 20 children who were not part of the main study. These children underwent a second evaluation one week later to verify the diagnosis of dental caries and the presence of plaque. To ensure consistency during re-evaluations, the same examiner, blinded to treatment allocation, conducted all follow-up clinical examinations. All re-evaluations were performed under standardized conditions, maintaining the same clinical setting, dental chair, and lighting (e.g., standard dental operating light) as during the baseline assessment.

The selection of children participating in the study began by contacting the Municipal Department of Education in Diamantina/MG to request authorization to screen in educational institutions in the city. Afterward, school principals were contacted, and parents received an authorization form for the children's clinical evaluation along with a questionnaire to assess dental caries risk. The school-based evaluation took place after the completed forms with parental authorization were received. The evaluated parameters included visible plaque, gingival inflammation, dental caries, restorations, missing teeth, and enamel defects. After the screening process, children and their parents were invited to participate in the study via an invitation letter.

Biofilm and gingivitis assessments were conducted using the Dichotomous Visible Plaque Index (VPI) (Ainamo et al., 1975), where code 0 indicated the absence of plaque and code 1 indicated the presence of visible plaque. The index value was expressed as a percentage by dividing the total number of surfaces with visible plaque by the total number of examined surfaces, then multiplying by 100 (Coutinho et al., 1997; Cardoso et al., 2000). The gingival bleeding index (dichotomous) was determined after gently probing the gingival sulcus on all teeth at four sites (buccal, lingual/palatal, mesial, and distal) using a spherical-tipped probe, with a 15-second interval. Each site was classified as code 0 for the absence of bleeding and code 1 for the presence of bleeding (Coutinho et al., 1997; Cardoso et al., 2000).

The selected children were invited to the Postgraduate Dental Clinic at the Federal University of Vales do Jequitinhonha and Mucuri, accompanied by their parents, for a clinical evaluation. After identifying plaque and gingivitis, dental prophylaxis was performed, followed by caries evaluation according to ICDAS criteria (Pitts N., "ICDAS", 2004). The children were randomly assigned via simple randomization (i.e., without stratification for risk level, age, or sex) to two prevention groups using random numbers generated by: Group 1 (Silver Diamine Fluoride - SDF) and Group 2 (Resin Sealant). Baseline characteristics, as shown in Table 1, confirmed a balanced distribution across groups for all sociodemographic and clinical variables.

For the SDF group, the tooth to be treated underwent prophylaxis with a Robinson brush and prophylactic paste, relative isolation using cheek retractors and cotton rolls, and soft tissue protection with petroleum jelly applied to the treatment area. The tooth was then dried with an air jet for 30 seconds, followed by the application of one drop of 30% Silver Diamine Fluoride (Cariestop 30%, Biodinâmica, Paraná, Brazil) to the pits and fissures using a disposable microbrush for three minutes. After this, the tooth was rinsed with water for one minute. This procedure constituted a single baseline application of SDF, specifically for preventive purposes, with no subsequent reapplications throughout the 24-month follow-up period. This protocol aimed to evaluate the primary preventive efficacy of a single SDF application compared with resin sealants.

In the Resin Sealant group, the selected tooth also underwent prophylaxis, relative isolation, and soft tissue protection. The occlusal surface was cleaned with a Robinson brush and pumice, then rinsed and dried with oil-free air. The pits and fissures were then etched for 30 seconds with Acigel 37% (SSWhite, Rio de Janeiro, RJ, Brazil), a 37% phosphoric acid gel, rinsed with water, and dried using a triple syringe. For this sealant material, an additional adhesive bonding agent was not used, as recommended by the manufacturer for direct application to etched enamel. The resin-based sealant Fluroshield (Dentsply, Rio de Janeiro, RJ, Brazil) was applied with a clinical probe (Duflex, Rio de Janeiro, RJ, Brazil) and light-cured for 40 seconds using an Ecel EC450 light-curing unit (Ecel, São Paulo, SP, Brazil), with an average irradiance of 600 mW/cm², periodically verified using a radiometer RD-7 (Ecel, São Paulo, SP, Brazil). Immediately after light curing, the sealant was visually inspected for complete coverage of the pits and fissures, marginal integrity, and absence of air bubbles. Any deficient applications were immediately reapplied. Occlusal contact was then checked with articulating paper and adjusted, if necessary, with rotary instruments.

After the procedure, regardless of the group to which the child was allocated, parents and children received oral hygiene guidance through the reading of informational booklets aloud, demonstrations on brushing techniques using a dental model, followed by supervised brushing by the child under the observation of their mothers, along with dietary advice, particularly regarding sugar consumption. These oral hygiene instructions were reinforced at each subsequent recall visit, throughout the 24-month follow-up period.

The following forms were used as data collection instruments: a sociodemographic and general and oral health data form; a child dietary diary; a treatment and evaluation form; a record sheet for other dental procedures; a questionnaire for assessing oral health-related quality of life (OHRQoL) in children (Child Perceptions Questionnaire, CPQ-8-10); a caregiver satisfaction scale; and a patient discomfort scale (Wong-Baker Faces Pain Rating Scale) (Barbosa et al., 2009; Wong et al., 1988).

The primary dependent variable was defined as the incidence of carious lesions on the occlusal surface of permanent first molars. Secondary dependent variables included: treatment type, sucrose consumption index, child's age, child's sex, brushing frequency, socioeconomic status, tooth type, surface type, presence of dental plaque, and presence of gingivitis.

Statistical analyses were performed using SPSS® for Windows® (Statistical Package for the Social Sciences Inc., Armonk, NY, USA) version 26. Exploratory data analyses provided frequencies, means, and standard deviations. Normality was assessed using the Shapiro-Wilk test. Quantitative data were analyzed using the Mann-Whitney test (for comparisons between groups) and the Wilcoxon test (for intra-group comparisons). Confidence intervals of 95% and a significance level of 5% were adopted. Loss to follow-up was addressed through a per-protocol analysis, which included only participants who completed the 24-month follow-up for the primary outcome. No imputation methods were used for missing data.

## Results

The initial study population consisted of 79 children. Of these, 11 (13.93%) were lost to follow-up. The main reasons for absence were changes in address or phone number, as well as refusal of care due to the pandemic period. Among the participating children, the majority were female (43; 63.2%) (Table 1). Most families had a monthly income of 1 to 2 minimum wages, with 32 (50%) participants having their mother as the primary caregiver (Table 1).

**Table 1:** Characteristics of participating children by group

	Sealant		SDF		
	n	%	n	%	p*
Sex					
Male	13	40.6	12	33.3	0.534
Female	19	59.4	24	66.7	
Mother's marital status					
Married	17	53.1	19	52.8	0.179
Common-law marriage	6	18.8	1	2.8	
Single	7	21.9	10	27.8	
Divorced	0	0.0	1	2.8	
Other	2	6.3	5	13.9	
Main caregiver occupation					
Employed	25	78.1	22	61.1	0.130
Unemployed	7	21.9	14	38.9	
Monthly income					
2 to <5 minimum wages	7	21,9	3	8.3	0.281
1 to <2 minimum wages	19	59.4	26	72.2	
<1 minimum wage	6	18.8	7	19.4	
Mother's education					
Higher education	9	28.1	5	13.9	0.345
Secondary	14	43.8	21	58.3	
Primary	9	28.1	9	25.0	
Does not know	0	0.0	1	2.8	
Father's education					
Higher education	4	12.5	3	8.3	0.734
Secondary	16	50.0	15	41.7	
Primary	10	31.3	14	38.9	
Does not know	2	6.3	4	11,1	
Type of housing					
Own	23	71.9	24	66.7	0.393
Granted	2	6.3	6	16.7	
Rented	7	21.9	6	16.7	
	Sealant		SDF		
	n	%	n	%	p*
Main caregiver of the child					
Mother	14	43.8	18	50.0	0.616
Father	1	3.1	5	13.9	
Grandparent	6	18.8	5	13.9	
Sibling	7	21.9	6	16.7	
Uncle/Aunt	1	3.1	1	2.8	
Nanny	2	6.3	1	2.8	
Other	1	3.1	0	0.0	
Child’s visits to the dentist					
Twice a year	8	25.0	0	0.0	0.070
Once a year	7	21.9	8	22.2	
Sometimes	10	31.3	8	22.2	
When in pain	3	9.4	7	19.4	
Never	4	12.5	7	19.4	
Last visit to the dentist					
Three months	3	9.4	0	0.0	0.070
Three to six months	5	15.6	1	2.8	
More than six months	20	62.5	30	83.3	
Never	3	9.4	2	8.3	
Where does the child receive dental care?					
UFVJM	20	64.5	27	75.0	0.604
Health center	7	22.6	5	13.9	
Private	4	12.9	4	11.1	
The caregiver helps with brushing.					
Regularly	9	28.1	3	8.3	0.102
Occasionally	10	31.3	14	38.9	
Never	13	40.6	19	52.8	
The caregiver noticed the first molar erupting.					
Yes	12	37.5	12	33.3	0.720
No	20	62.5	24	66.7	
The child brushes their teeth once per day.					
More than twice	22	68.8	18	50.0	0.087
Twice	9	28.1	11	30.6	
Once	1	3.1	7	19.4	
Sometimes	0	0.0	0	0.0	
A child uses dental floss.					
More than twice	2	6.3	2	5.6	0.131
Twice	1	3.1	2	5.6	
Once	3	9.4	3	8.3	
Sometimes	19	59.4	11	30.6	
Never	7	21.9	18	50.0	

*Chi-square test

Children who received SDF showed a significant reduction in VPI after 24 months compared with baseline (p = 0.027). The parameters of bleeding on probing, procedure time, and patient discomfort did not show statistically significant differences between the two evaluation points in either group (Table 2).

**Table 2:** Distribution of children according to the treatment received (TI) at baseline (T0) and at the second evaluation.

	VPI inicial	VPI after	p*
	24 months		
Sealant	29.42 (23.96)	34.63 (31,50)	0.731
SDF	39.35 (28.19)	28.47 (25,57)	0.027
p-value*	0.158	0.635	
	BOP inicial	BOP after	p*
	24 months		
Sealant	15.36 (21.54)	15.86 (16.18)	0.600
SDF	9.83 (15.87)	12.03 (16.86)	0.192
p-value*	0.196	0.131	
	CPQ inicial	CPQ after	p*
	24 months		
Sealant	19.87 (7.11)	22.63 (7.80)	0.001
SDF	18.80 (7.81)	21.00 (7.12)	0.009
p-value*	0.460	0.376	
Processing Time			
Sealant	20.27 (6.28)		
SDF	18.70 (6.74)		
p-value*	0.195		
Wong Becker scale			
Sealant	0.31 (0.53)		
SDF	0.53 (0.77)		
p-value*	0.321		

*Mann-Whitney Test* *Wilcochon Test*

Regarding the CPQ index, which measures quality of life, scores increased in the second evaluation in both groups. They were statistically significant, indicating a decrease in children's quality of life across treatment groups (Table 2).

There was significant darkening of the teeth treated with SDF after 24 months (<0.001) (Table 3). Patients did not report pain for any of the materials studied, and in both procedures, the children exhibited upbeat or definitely positive behavior (Table 3).

The SDF group showed a higher success rate (88.6%) than the sealant group (80.4%); however, there was no significant difference in the distribution of caries presence between the groups after 24 months (p = 0.086) (Table 3).

**Table 3:** Comparison of initial and final characteristics between participants who followed

	Sealant		SDF		
	n	%	n	%	p*
Initial darkening					
Absent	19	86.4	11	30.6	<0.001
Present	03	13.6	25	69.4	
Initial pain					
Absent	22	100.0	36	100.0	-
Present	0	0.0	0	0,0	
Changes in the initial mucosa					
None	22	100.0	36	100.0	-
Darkening after 24 months					
Absent	21	95.5	11	30.6	<0.001
Present	1	4.5	25	69.4	
Pain after 24 months					
Absent	22	100.0	36	100.0	-
Present	0	0	0	0	
Changes in the mucosa after 24 months					
None	22	100.0	35	97.2	0.430
Swollen	0	0.0	1	2.8	
Child's behavior					
Definitely positive	6	18.8	11	30.6	0.262
Positive	26	81.3	25	69.4	
Treatment success					
Without cavities	82	80.4	109	88.6	0.086
With cavities	20	19.6	14	11.4	

## Discussion

The present study compared the preventive effects of silver diamine fluoride (SDF) and resin-based sealants on the development of occlusal caries in the permanent first molars of children at high caries risk over 24 months. Although both interventions demonstrated satisfactory preventive outcomes, the SDF group had a higher percentage of caries-free teeth (88.6%) than the sealant group (80.4%), with no statistically significant difference between the two. These findings suggest that SDF may be as effective as conventional resin sealants in preventing new occlusal lesions in permanent molars, supporting its potential use as a simple, low-cost, and minimally invasive alternative, especially in community or school-based prevention programs where traditional sealant placement may be limited by moisture control or access to dental equipment ^20^.

SDF is a liquid compound applied topically, capable of halting the progression of carious lesions without the need to remove infected soft tissues and with minimal moisture control requirements ^21^. These features make it advantageous for implementation in public oral health programs and even in community projects aimed at preventing dental caries in school-aged children, especially those living in areas with limited access to dental services ^22^.

Resin-based sealants have a well-documented clinical use for preventing caries in the pits and fissures of permanent teeth. They are considered the first choice for this purpose, with a reduction in caries activity of up to 76% reported by the American Academy of Pediatric Dentistry ^23^. A meta-analysis conducted by Llodra et al. (1993) demonstrated a preventive fraction of 70% over a 36-month follow-up period, further emphasizing the effectiveness of this preventive approach.

Resin-based sealants are hydrophobic, which makes them more sensitive to moisture contamination, but they also provide better adhesion to tooth structures and fluoride release ^25^. While they offer pleasing aesthetics, the clinical success of sealants depends heavily on the application technique, moisture control during the procedure, and the operator's skill. In this study, the method was applied under ideal conditions, yielding an excellent retention rate after a 2-year follow-up.

The ability of silver diamine fluoride to perform effectively in hostile environments is of great importance to dentistry. In this study, this property was validated, as most children undergoing treatment do not practice supervised or adequate oral hygiene or regularly visit the dentist for routine check-ups, thereby increasing their risk of developing carious lesions. This reality may also contribute to challenges in patient management, as it hinders familiarity with the dental environment and may compromise the quality of sealant application.

The reduction in plaque index observed in the group treated with silver diamine fluoride (SDF) can be attributed to the material's antimicrobial properties. Silver diamine fluoride can inhibit the growth of bacteria in dental biofilm by acting directly on them. This results in a significant reduction in the quantity of bacteria that play a crucial role in plaque formation ^26^. In summary, SDF acts as an effective antimicrobial agent, helping control the oral microbiota and, consequently, reducing dental plaque development.

Although silver diamine fluoride is effective in preventing caries, the potential for tooth staining is an important consideration. The silver in its composition can react with compounds on the tooth surface, leading to dark stains. Additionally, SDF can interact with other substances in the oral cavity, such as sulfur from food or saliva, forming silver sulfide complexes that contribute to tooth discoloration ^27^.

The application technique also plays a crucial role in this regard, as excess SDF may accumulate on tooth surfaces and cause the same effect. This factor may explain the staining observed in the final clinical evaluation of this study and aligns with the literature on the side effects of SDF application.

Regarding the time required for the procedure, it is generally expected that SDF takes less time to apply than sealants (Silva Ribeiro Junior et al., 2023). However, in this study, no difference was observed between the groups regarding procedural time. This occurred because the procedures were standardized, with identical steps for application, which might differ in a real clinical setting.

It is important to note that the unpleasant taste of SDF can cause discomfort for patients (Ruff et al., 2024). Thus, although SDF is an effective option for caries prevention, dental professionals should consider potential side effects when recommending it. During this study, 50% of patients who received SDF reported an unpleasant taste. This side effect can be minimized with improved relative isolation during product application.

Another concern with SDF, particularly with commercially available Brazilian products, is the ideal concentration and fluoride content (Soares-Yoshikawa et al., 2020). In this study, a 30% concentration was used, as it is the most readily available commercially in Brazil. However, most studies use a 38% concentration, which may yield different clinical outcomes. Future clinical and laboratory studies are needed to investigate this issue further.

The protective capacity of silver diamine fluoride (SDF) against dental caries is explained by its ability to remineralize carious lesions by recovering calcium and phosphate from saliva, reducing dentin collagen degradation, and inhibiting collagenases such as MMPs and cathepsins ^28^. In the present study, no statistically significant difference was observed between the groups in dental caries development after 24 months (p = 0.085). However, when comparing incidence rates, the SDF group drastically reduced the number of active lesions, whose chemical and biological caries-protective properties readily explain this reduction. In studies with larger sample sizes, this difference might become statistically significant.

Regarding quality of life, the present study showed a reduction in CPQ scores after both treatments. This unexpected finding may be attributed to external factors related to the COVID-19 pandemic that occurred during the study period, which may have biased the results. Several recent studies have demonstrated a decline in children's quality of life associated with the pandemic (Orban et al., 2024; Pang et al., 2023; Yasmin et al., 2021), supporting this possibility.

Most clinical studies investigating SDF use focus on the treatment of primary teeth. To date, five studies have addressed the use of SDF in permanent teeth (Ruff et al., 2023; Monse et al., 2012; Liu et al., 2012; Llodra et al., 2005; Antonioni et al., 2019). Among these, only the studies by Llodra et al. (2005) and Liu et al. (2012) explored the use of SDF as a preventive approach in non-carious permanent teeth, a proposal similar to the one presented in the present study.

In a study conducted by Liu et al. (2012), the efficacy of SDF in preventing caries in pits and fissures after two years was reported to be 87.8%. The sealant-treated group showed an efficacy of 92%, with no statistically significant difference between the two groups, closely resembling the success rate found in our study (80.4% for sealants and 88.6% for SDF). However, it is essential to note that, in Liu’s study, the treatments were administered to schoolchildren in real-world settings. In contrast, our study took place in a controlled dental office environment. The difference in application settings may have negatively impacted the retention rate of resin-based sealants, as observed in previous studies.

The study by Llodra et al. (2005) employed a comprehensive approach, including both permanent and primary teeth in its sample, and compared SDF with an untreated control. The preventive fraction observed in the SDF-treated group was 65% for first permanent molars, a value similar to that of the present study.

Although the SDF group showed a higher percentage of caries-free teeth (88.6%) than the sealant group (80.4%), the difference was not statistically significant and may be considered clinically modest. From a public health perspective, such a slight variation suggests that both interventions provide comparable protection against occlusal caries in permanent molars. Considering that SDF is less technique-sensitive, requires minimal equipment, and is considerably more affordable, its similar effectiveness to resin sealants highlights its potential as a practical and equitable preventive strategy, particularly in community and school-based programs where access to conventional dental care is limited. Therefore, even in the absence of a statistically significant difference, the findings have meaningful implications for expanding preventive coverage in underserved populations.

In regions or communities where dental resources and services are readily accessible, resin-based sealants may be the preferred option for preventing caries in schoolchildren. However, in less developed areas where economic and resource constraints are more prevalent, applying SDF to the pits and fissures of molars may be a viable alternative to resin-based sealants.

This study presents some limitations that should be considered. The controlled environment in which the applications were performed may have influenced the results, particularly regarding the retention rate of resin sealants, which may differ from real clinical situations. Another relevant point was the occurrence of tooth staining and reports of an unpleasant taste, which, although expected, may affect treatment acceptance. Future studies should explore different concentrations of SDF available in the Brazilian market, as well as alternatives to minimize adverse effects. It is also necessary to investigate the efficacy of SDF in larger populations and less controlled clinical settings, as well as to assess its long-term impact on quality of life and oral health. Ultimately, further research should consider direct comparisons with new formulations of resin sealants, thereby expanding the scientific evidence to inform more robust clinical decisions.

## Conclusion

The SDF proved to be an effective alternative to resin sealants for preventing pit-and-fissure caries in first permanent molars after a 2-year follow-up.
